# Fructose and Pectin Detection in Fruit-Based Food Products by Surface-Enhanced Raman Spectroscopy

**DOI:** 10.3390/s17040839

**Published:** 2017-04-11

**Authors:** Carlo Camerlingo, Marianna Portaccio, Rosarita Tatè, Maria Lepore, Ines Delfino

**Affiliations:** 1 CNR-SPIN, Istituto Superconduttori, Materiali Innovativi e Dispositivi, 80078 Pozzuoli (Na), Italy; carlo.camerlingo@spin.cnr.it; 2 Dipartimento di Medicina Sperimentale, Università della Campania “Luigi Vanvitelli”, 80138 Naples, Italy; marianna.portaccio@unicampania.it; 3 Institute of Genetics and Biophysics—ABT, CNR, 80131 Naples, Italy; rosarita.tate@igb.cnr.it; 4 Dipartimento di Scienze Ecologiche e Biologiche, Università della Tuscia, 01100 Viterbo, Italy

**Keywords:** surface enhanced Raman spectroscopy (SERS), gold nanoparticles (GNPs), commercial fruit juice and fruit-based preparations, pectin, fructose

## Abstract

Surface-Enhanced Raman Spectroscopy (SERS) enables the investigation of samples with weak specific Raman signals, such as opaque samples, including fruit juices and pulp. In this paper, biological apple juices and apple/pear pulp have been studied in order to evidence the presence of fructose and pectin, which are components of great relevance for quality assessment of these kinds of products. In order to perform SERS measurements a low-cost home-made substrate consisting of a glass slide decorated with 30-nm-sized gold nanoparticles has been designed and used. By employing a conventional micro-Raman spectroscopy set-up and a suitable data treatment based on “wavelet” denoising algorithms and background subtraction, spectra of pectin and fructose with clear Raman features have been obtained. The results have confirmed the potential of SERS in the food industry for product characterization, also considering the low-cost and the relative ease of the fabrication process of the employed SERS substrate.

## 1. Introduction

Over the past few decades Surface-Enhanced Raman Spectroscopy (SERS) has been fruitfully used for investigating materials with weak Raman signals and for the detection and study of various molecules down to the single-molecule level [1,2,3,4,5]. SERS combines the advantages of Raman spectroscopy such as its high specificity, and the ability to identify a given molecular species in the presence of many other chemicals, with the relevant Raman signal enhancement enabled by nanosized metallic materials. This enhancing effect results from the electromagnetic field enhancement induced by the localized surface plasmon resonance (SPR) in nanosized metallic materials and from the chemical enhancement caused by the charge interactions between the probe molecules and metal nanostructures induced by light [6,7]. Among many nanomaterials, gold nanoparticles (GNPs), both spherical and with anisotropic morphologies, and their colloidal dispersions, have attracted great interest for SERS applications due to their unique properties, such as the availability of fabrication processes insuring high control and homogeneity of the particle size, a large surface area to volume ratio, the high reactivity to living cells and the stability over long times and high temperatures [8,9,10,11,12]. Thanks to the wide use of GNPs and GNP-based substrates, SERS is growing in popularity also in the field of food production and quality control [13,14,15,16,17,18,19,20,21]. Recently, it has been employed for detecting trace amounts of the fungicide thiram on fruit peels [15,18,19], of pesticides in apples and tomatoes [20], and of vitamins in cereals [21]. Additionally, SERS holds potential for the realization of methods and devices enabling a simple, low-cost and low time-consuming evaluation of the content of commercial food industry products such as fruit juices, soft drinks and so on. In fact, on the one hand it has been shown that Raman spectroscopy is a valuable tool for characterizing such media with no preliminary sample preparation procedure [22,23,24,25,26]. On the other hand, the use of SERS enables also the investigation of the samples of interest for food industry having a low Raman signal level, such as olive oil samples and drinks [27,28,29]. A crucial aspect for using SERS in industrial applications is the availability of simple and low-cost SERS substrates that can be routinely used even on production lines. One possible way to face this aspect is the use of home-made GNPs-based SERS substrates as reported in the present work in which a well-assessed and widely employed GNPs preparation method is adopted [30,31,32]. Using this kind of substrate SERS spectra from apple and apple/pear based commercial biological products have been obtained and the fingerprints of fructose and pectin have been observed and discussed. The control of these components is of great importance from industrial point of view in order to assess the quality and reproducibility of the products and their nutritional factors [33]. Fructose and pectin content are, respectively, related to nutritional properties and turbidity of the products [34,35]. It is worth noting that the biochemical assays generally employed to quantify the above-mentioned substances are time consuming and require the use of a specific chemical reagent for each component [36,37,38]. Then the availability of other (low-time and chemical consuming) methods for detecting them is important in food industry.

The results reported here show that the proposed method allows one to obtain SERS spectra with clear specific fingerprints of the components of interest. Notably, the results have been obtained without any sample pre-treatment and by using conventional micro-Raman (µ-RS) equipment. Moreover, the used automatic elaboration of the spectral data has allowed us to perform a fast sampling of the products. The results indicate that SERS technique can be employed for efficiently studying a wide variety of food industry products overcoming the limits induced by sample turbidity and light-scattering properties on the Raman response.

## 2. Materials and Methods

### 2.1. Materials

For GNP fabrication hydrogen tetrachloroaurate (HAuCl_4_) and trisodium citrate were used. These chemical products were purchased from Sigma-Aldrich (St. Louis, MO, USA) and were used as received.

The composition of a fruit juice depends on many factors as the fruit variety, origin and growing conditions, its quality and the processing and storage procedures that it has undergone. Excluding water, the major components of fruit juices are sugars in a concentration of about 120 mg/mL, including mainly fructose but also sucrose and glucose, pectin (1–5 mg/mL), starch (0.5–5 mg/mL) and malic acid (3–7 mg/mL) [22]. Furthermore, they contain important components as vitamins, polyphenols and proteins in smaller quantity [22].

In the present work, commercial biological fruit juice and pulp preparation were investigated to detect the presence of fructose and pectin. As said before, fructose and pectin content is related to nutritional properties and turbidity of the products, respectively. The commercial juice was from biological cultivation and production, according to the producer, and appears more turbid than the clarified juices previously investigated (not from biological production) by some of the authors [22]. The commercial smashed apple/pear pulp preparation (composition: of 50% of apple and 50% of pear, without additives) was investigated to prove the affordability of the proposed method also for the characterization of highly turbid samples. Apple juice and commercial smashed pulp of apple and pear were used without further treatment. Information about the juice and pulp producers are available on request. As a reference, pure crystalline fructose (Startingline SpA, Nerviano Milan, Italy) was considered.

### 2.2. Nanoparticle Preparation and Characterization

GNP preparations were obtained by the conventional citrate reduction method [30,31]. A 0.01% HAuCl_4_ solution was reduced by 1% sodium citrate with vigorous stirring at near boiling temperature. The amount of sodium citrate was defined in order to obtain GNPs with an expected diameter of about 30 nm since preliminary measurements have shown that this is a good option for SERS applications [17]. Particle size and preparation stability of the resulting nanoparticles were investigated by means of absorption spectroscopy, Transmission Electron Microscope (TEM), and Dynamic Light Scattering (DLS). In particular, absorption spectroscopy was used to get information on the plasmon resonance peak and to study the stability of the preparations [39,40]. TEM and DLS were employed to estimate the size of the GNPs. Raman signal due to GNP preparations was also evaluated (see Section 2.4 for details about the used set-up and measurement protocol).

Absorption spectra of the prepared GNPs suspensions were recorded by a two-beam UV–VIS spectrophotometer (LS25, Perkin Elmer, Waltham, MA, USA). All the spectra were collected over the range of 200–800 nm with 2.0 nm resolution. TEM images were obtained by a JEOL JEM-1011 TEM (JEOL, Tokyo, Japan) equipped with a thermionic tungsten filament and operated at an acceleration voltage of 100 kV. Images were taken using a cooled slow-scan CCD camera (Morada, Munster, Germany, 3783 × 2672 pixels) and micrographs were taken with iTEM software (Olympus Soft Imaging System GmbH, Munster, Germany). Particle sizing by DLS was performed using a Zetasizer Nano Series and DLS system (Malvern Instruments Ltd., Malvern, UK).

### 2.3. Sample Preparation

A small drop of the colloidal suspension of GNPs was placed on a microscope glass and left to dry at room temperature. After about one hour the solvent (mainly water) evaporated leaving the GNPs dispersed on the glass surface into a limited area. A small amount of the sample to be analyzed (apple juice or smashed apple/pear pulp) was dropped on this area and right after the SERS measurements were performed. Furthermore, aqueous solutions of fructose at different concentrations were prepared. Starting from saturated fructose/water solution, with a nominal concentration of fructose of 730 mg/mL, diluted solutions were prepared with concentration varying in the 20–50 mg/mL range, within the range of fructose concentration in commercial juices [33]. For comparison µ-RS measurements were performed on samples placed on a bare microscope glass.

### 2.4. Micro-Raman Spectroscopy and SERS Measurements

The µ-RS and SERS measurements were performed by using a Jobin-Yvon system from Horiba Scientific ISA (Edison, NJ, USA), equipped with a TriAx 180 monochromator, a liquid nitrogen cooled charge-coupled detector and a 1800 grooves/mm grating (final spectral resolution: 4 cm^−1^). The spectra were recorded in air at room temperature using a 17 mW He-Ne laser source (wavelength 632.8 nm). Accumulation times in the 30–300 s range were used. The laser beam was focused to a 2-µm spot size on the sample through an Olympus microscope equipped with 50× optical objective.

### 2.5. Data Analysis

The Raman spectra collected from complex media typically show a smeared background signal. In order to enhance the signal readability and attenuate background and noise components, an automatic numerical treatment based on the wavelet algorithm was used [41]. This method allows a reliable quantitative evaluation of spectral details also in the case of very weak signals, and it had been applied successfully to the Raman analysis of human single cell [42], and blood serum [43]. Briefly, the spectrum was decomposed in terms of the sum of different wavenumber-scaled elementary functions (named wavelets) and a hierarchical representation of the spectrum was thus obtained. Starting from the decomposed signal the spectrum was reconstructed removing low and high frequency components due to background and non-correlated noise respectively. MATLAB 6.5 program (by Math Works Inc., Natick, MA, USA) was used for wavelet analysis with wavelet family of biorthogonal functions “bior6.8”. The decomposition of the signal was performed up to the eighth level. Subsequently the signal was reconstructed by employing only detail components from 5th to 8th level.

In order to determine the basic vibrational modes that contribute to the Raman signal, the spectra were analyzed in terms of convoluted Lorentzian functions by using a best-fit peak-fitting routine of GRAMS/AI program (2001, Thermo Fisher Scientific, Waltham, MA, USA), based on the Levenberg-Marquardt nonlinear least-square method. The main peaks of the spectrum were manually selected in order to define the starting conditions for the best-fit procedure. The best-fit was then performed to determine the convolution peaks with optimized intensity, position and width. Its performance was evaluated by means of the χ^2^ parameter. For further details see [23].

## 3. Results

### 3.1. Nanoparticle Characterization

**Figure 1:**
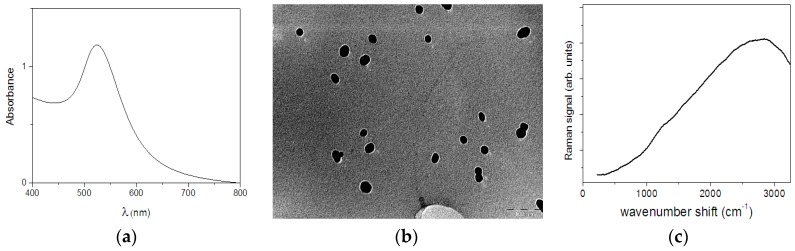
(**a**) Absorption spectra; (**b**) TEM image (with scale bar = 100 nm) and (**c**) Raman spectrum of GNP preparation (see text for details).

In Figure 1a the optical absorption spectrum of the GNPs suspension is shown. It is characterized by the presence of a single broad peak in the 400–800 nm range, as expected for colloidal dispersions of spherical metallic nanoparticles [39,40]. In fact, the observed feature represents the SPR absorption band of the metal nanoparticles that provide valuable information on the size, structure and aggregation of GNPs. According to [39], a mean GNP diameter of 30 ± 4 nm is estimated using the observed SPR band peak position.

The absorption spectra remain unchanged for long time suggesting a stability of the preparation of more than 6 months (data not shown). A representative TEM image of the GNPs preparation is shown in Figure 1b, where mainly single spherical GNPs are observed. It confirms the average size estimated by the SPR absorption analysis even if a bigger dispersion is evinced. TEM results are in agreement with those from DLS that have provided a mean diameter for the GNPs of 27 ± 7 nm.

The Raman spectrum of the dried GNP suspension is reported in Figure 1c for the 200–3200 cm^−1^ range. A featureless signal with a broad peak located at around 2800 cm^−1^ is observed. In particular, no specific features due to the GNPs are present in the 200–1800 cm^−1^ range that is the region of interest for the main Raman vibrational modes expected from fruit juice and pulp. This allows us to state that the dried GNPs suspension can be used as substrate for SERS enhancement since it will not give any disturbing signals in the region of interest.

### 3.2. Raman and SERS Measurements

**Figure 2:**
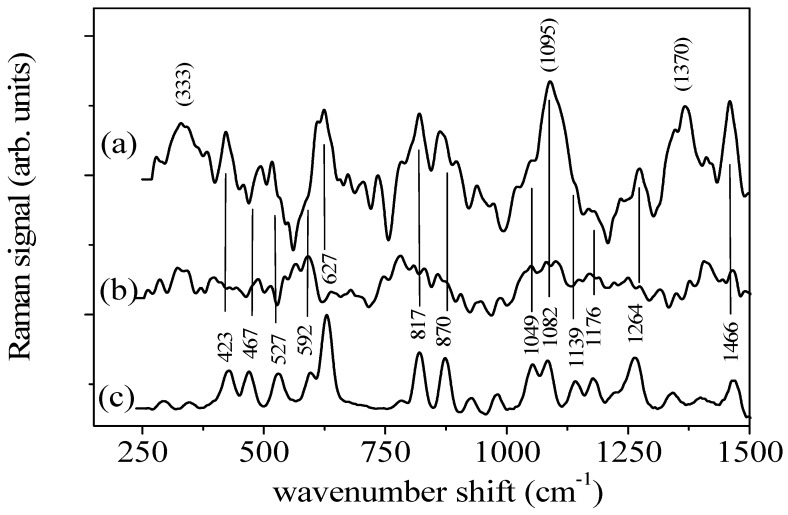
(**a**) SERS spectrum of the saturated aqueous solution of fructose; (**b**) Conventional Raman spectrum of the same fructose solution; (**c**) Raman spectrum of crystalline fructose. The position of the main modes of crystalline fructose are labelled. Positions of the features of SERS spectrum of fructose solution with unexpected strong intensity (at 333 cm^−1^, 1095 cm^−1^ and 1370 cm^−1^) are reported in brackets.

The ability of the GNPs-based substrate to produce Raman enhancement in the spectral region and for the components of interest has been studied by using fructose as analytical probe since it has well established Raman features. In Figure 2 the Raman spectra of a saturated aqueous solution of fructose as obtained by using the GNPs-based substrate (Figure 2a) and by placing the solution on a bare glass slide (Figure 2b) are shown.

The first spectrum features clear sharp peaks while the other one has only broad low-intensity bands, thus suggesting that the use of the substrate increases the intensity of the peaks with respect to conventional μ-RS. Furthermore, the peaks observed in the SERS spectrum can be identified as the main modes of fructose by comparing the data with the Raman spectrum of crystalline fructose, also shown in Figure 2c. The positions of the main modes of crystalline fructose are labelled in the same figure and listed with their assignments in Table 1, where their relative intensities are also reported (the intensity of the strong mode at 627 cm^−1^ is taken as reference). The observed modes have characteristics that are in good agreement with those observed in the Raman spectra of fructose aqueous solutions reported in [44] even if unexpected high intensity and shift in the position are observed for the modes centered at 333, 1095 and 1370 cm^−1^, assigned to C-O-C and C-O-H bending and CH_2_ wagging, respectively [44]. In particular, the band centered at 330 cm^−1^ shows a large wavenumber shift decrease in comparison to the expected position (344 cm^−1^) and an intensity enhancement. The feature located at 1095 cm^−1^ in the SERS spectrum is shifted to higher wavenumber shifts and has a larger intensity as compared to its counterpart observed in the Raman spectrum of crystalline fructose. Moreover the 1370 cm^−1^ mode is detectable only in the SERS spectrum. An unexpected intensity enhancement is detected also for the peaks due to pyranose C-C-C and to CH_2_ bending modes observed at 424 and 1460 cm^−1^, respectively, in the spectrum obtained by using the SERS substrate. In addition, it has been observed that the feature due to the C-C-O bending (622-cm^−1^ peak observed in the fructose SERS spectrum) becomes sharper when the water content increases as evidenced from the SERS spectra of the other fructose solutions examined (data not shown), whose concentrations fall in the industrially relevant range (20–50 mg/mL). As well-known some differences in the positions and intensity of fructose modes can occur depending on the SERS effect itself [1,2,7] and on the water content [45].

The discussed results prove that the home-made GNPs-decorated glass slide is a suitable SERS substrate. Accordingly, it was employed to investigate the selected commercial fruit juice and pulp samples.

**Figure 3:**
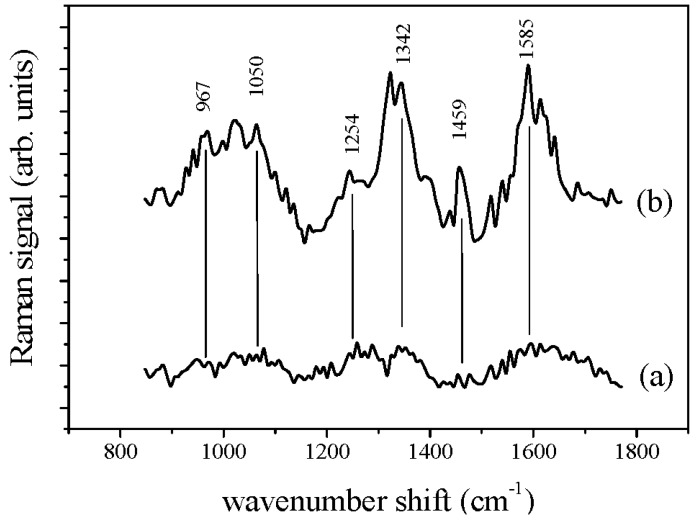
Raman spectra of apple juice after background signal subtraction and denoising procedure, obtained from bare juice drop (**a**), and from juice drop on the home-made GNPs-based SERS substrate (**b**). The spectra are arbitrarily shifted along the *y*-axis.

In Figure 3 the Raman spectra of apple juice after background signal subtraction and denoising procedure [41], obtained from bare juice drop (Figure 3a) and from juice drop on the home-made GNPs- based SERS substrate (Figure 3b) are shown.

It is evident that clear features are observed only in the spectrum from juice drop on the GNPs-based SERS substrate. Hence, differently from the case of the clarified apple juices previously investigated by some of the authors [22], in the present a significant Raman spectrum cannot be obtained by conventional µ-RS. Hence, only the SERS spectrum will be further considered. The most important features of SERS spectrum are reported in Table 2 together with their tentative assignment to fructose and pectin.

**Figure 4:**
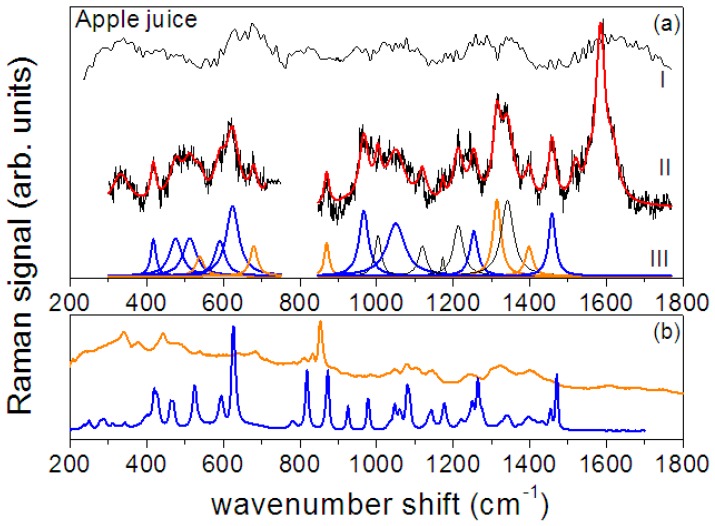
(**a**) Biological apple juice: the Raman spectrum (I) is compared with SERS response (black line) (II) and spectrum deconvolution in Lorentzian functions (III). Red line represents the convolution of component peaks. In orange are indicated the peaks assigned to pectin and in blue those assigned to fructose; (**b**) Spectra of amorphous dry fructose (blue line) and of amidate lime/lemon pectin (orange line) [48].

In addition, details and analysis of the SERS spectrum of biological apple juice are shown in Figure 4. In particular, in Figure 4a a zoom of the spectrum for the 300–750 cm^−1^ and 850–1750 cm^−1^ ranges is reported along with the results of the described deconvolution procedure in terms of Lorentzian curves (red line). Regarding the 350–750 cm^−1^ range, seven Lorentzian modes are outlined. They can be reasonably assigned to fructose and pectin [22]. In fact, the five peaks at 417, 475, 512, 591, 624 cm^−1^ (blue Lorentzian curves in Figure 4a) are located at positions close to peaks previously found in the fructose Raman spectrum (blue curve in Figure 4b), namely 423, 467, 527, 592, 627 cm^−1^. These overall correspondences and the above-discussed expected shift in the positions of the peaks occurring when SERS spectrum is detected [1,2,7] give us confidence that the observed features can be assigned to fructose. The two peaks observed at 538 and 679 cm^−1^ (orange Lorentzian curves in Figure 4a) can be assigned to pectin [47] and indicate the presence of pectin in the juice. In fact, similar features can be observed in the typical Raman spectrum of pectin (amidate lime/lemon pectin) that is also reported in Figure 4b as an orange line [48] for comparison.

In the 850–1700 cm^−1^ range 17 modes are outlined by the deconvolution procedure. As before, the majority finds a good correspondence with peaks of fructose and pectin (see their spectra shown in Figure 4b). For the sake of clarity all the observed peaks are reported in Table 2 along with their tentative assignment to pectin or fructose.

The spectrum in the 850–1700 cm^−1^ range is dominated by three main broad modes located at about 1004, 1342 and 1585 cm^−1^. The peak at 1004 cm^−1^ can be assigned to methyl component rocking modes, typically present in protein and carotene. This mode is typically found in the Raman spectra of flavonols [49].

Apple fruits, and in particular apple peel, contain flavonols, mainly found in rutin and other quercetin glycosides, thus we can tentatively assign this mode to the flavonol content [50]. The flavonol content depends on the solar exposition of the fruit during the growth, thus an assessment of its presence provides an interesting information on the quality of the processed fruits.

**Figure 5:**
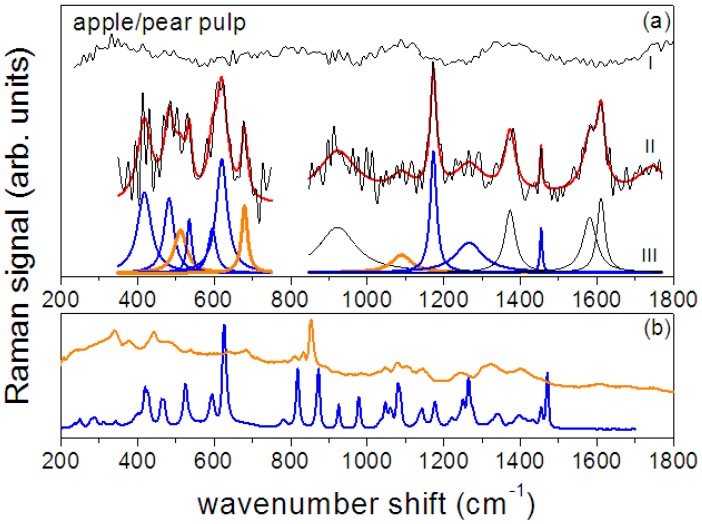
(**a**) Apple/pear smashed pulp: the Raman spectrum (I) is compared with SERS response (black line) (II) and spectrum deconvolution in Lorentzian functions (III). Red line represents the convolution of component peaks. In orange are indicated the peaks assigned to pectin and in blue those assigned to fructose. (**b**) Spectra of amorphous dry fructose (blue line) and of amidate lime/lemon pectin (orange) [48].

The spectra of the smashed apple/pear pulp as resulting after the data analysis procedure are reported in Figure 5a for the bare pulp drop on bare glass and for the pulp drop on the SERS substrate. The reference spectra of fructose (blue line) and pectin (orange line) are also shown for comparison (Figure 5b). Also in this case, the Raman signal detected from the bare pulp is relatively weak and no clear Raman peaks can be distinguished. On the contrary, when the signal is detected from the pulp dropped on the GNPs-based substrate a spectrum with well-defined features is obtained confirming the enhancement ability of the SERS substrate.

The SERS spectrum of apple/pear pulp shown in Figure 5b has been deconvoluted in terms of Lorentzian functions; the resulting curves are displayed in the figure (blue peaks are assigned to fructose and orange ones to pectin) and their positions are reported in Table 2. Similar to what was found for apple juice, in the 350–750 cm^−1^ range the fit procedure allows the determination of five main peaks (417, 482, 512, 595, 620 cm^−1^, blue Lorentzian curves) that can be assigned to fructose [46] and two (at 535 and 679 cm^−1^, orange Lorentzian curves) that can be assigned to pectin thus revealing the pectin content. Their intensity is slightly larger than that found in biological apple juice, as expected in an untreated fruit pulp. At higher wavenumber shifts the apple/pear SERS spectrum is dominated by three intense peaks at about 1173, 1373 and 1611 cm^−1^. In this case too, the contribution of pectin to the spectrum can be envisaged by the comparison of the spectrum with the reference signal of Figure 5b.

## 4. Conclusions

The reported results show that SERS spectra of commercial biological apple juice and pear/apple smashed pulp can be obtained, even in the cases when a low Raman signal or an excess of light dispersion of the samples drastically limit the use of conventional Raman assessment methods. SERS measurements can be performed using a conventional micro-Raman equipment and a low-cost and easy-to-prepare GNPs-based substrate without major financial or technological efforts. The great advantages peculiar of nanotechnology can find, in this way, a profitable use in the industrial field, facilitating the employment of Raman spectroscopy and extending its application limits. This is particularly important in food industry and in particular in the industries concerning fruit conservation and preparation, where a precise and reliable content control is a mandatory task in order to insure safety, quality and reliability of the product. In particular, we demonstrated in this paper the potentialities of SERS in the detection of fructose and pectin in commercial apple juices and in pear/apple pulp preparations.

## Figures and Tables

**Table 1:** Experimental positions and relative intensities, as obtained by the deconvolution procedure, of the vibrational modes observed in the Raman spectrum of crystalline fructose and in SERS spectrum of fructose aqueous solutions along with their tentative assignment made according to Ref. [44].

Crystalline Fructose		Fructose in Aqueous Solution		Assignment
Position (cm^−1^)	Relative Intensity (%)	Position (cm^−1^)	Relative Intensity (%)	
290	10	-	-	-
344	8	333	81	C-O-C bending
423	39	424	81	C-C-C bending (pyranose)
467	39	492	36	C-C-C bending (furanose)
527	37	517	42	C-C-O bending (pyranose)
592	32	-	-	-
627	100 ^a^	622	100 ^a^	C-C-O bending
-	-	678	20	C-C-O bending (furanose)
778	9	-	-	-
817	68	815	81	C-C stretching (pyranose)
870	63	872	66	C-C stretching (furanose)
923	19	940	19	C-C-H bending (furanose)
977	24	-	-	C-C-H bending (pyranose)
1049	46	1058	20	C-O stretching
1082	51	1095	128	C-O-H bending
1139	31	-	-	C-O stretching (pyranose)
1176	33	-	-	C-O stretching (furanose)
1241	25	-	-	-
1264	44	1272	43	CH_2_ twisting
1338	18	1340	52	-
-	-	1370	97	CH_2_ wagging
1404	18	1412	31	-
1466	38	1460	100	CH_2_ bending

^a^ Taken as intensity reference.

**Table 2:** Positions of the modes outlined by the deconvolution procedure in SERS spectra of commercial biological apple juice and of apple/pear smashed pulp and their tentative assignment to fructose or/and pectin [44,46,47].

Apple Juice—Peak Position (cm^−1^)	Apple/Pear Pulp—Peak Position (cm^−1^)	Fructose	Pectin
417	417	X	
475	482	X	
512	512	X	
538	535		X
591	595	X	
624	620	X	
679	679		X
730		X	
870		X	X
918	922	X	
967		X	
1004			
1050		X	
	1090	X	
1120			
1173	1173	X	
1213			
1254	1266	X	
1315			
1342		X	
	1373	X	
1398			
1458	1455	X	
1520			
1585	1581		
1554			
1617	1611		
	1746		
